# Arsenic Exposure and Type 2 Diabetes: A Systematic Review of the Experimental and Epidemiologic Evidence

**DOI:** 10.1289/ehp.8551

**Published:** 2005-12-15

**Authors:** Ana Navas-Acien, Ellen K. Silbergeld, Robin A. Streeter, Jeanne M. Clark, Thomas A. Burke, Eliseo Guallar

**Affiliations:** 1 Department of Epidemiology, Johns Hopkins University Bloomberg School of Public Health,; 2 Welch Center for Prevention, Epidemiology and Clinical Research, Johns Hopkins Medical Institutions,; 3 Johns Hopkins Center for Excellence in Environmental Public Health Tracking, Johns Hopkins University Bloomberg School of Public Health,; 4 Department of Environmental Health Sciences, Johns Hopkins University Bloomberg School of Public Health,; 5 Department of Medicine, Johns Hopkins School of Medicine and; 6 Department of Health Policy and Management, Johns Hopkins University Bloomberg School of Public Health, Baltimore, Maryland, USA

**Keywords:** arsenic, diabetes, glucose metabolism, meta-analysis, systematic review

## Abstract

Chronic arsenic exposure has been suggested to contribute to diabetes development. We performed a systematic review of the experimental and epidemiologic evidence on the association of arsenic and type 2 diabetes. We identified 19 *in vitro* studies of arsenic and glucose metabolism. Five studies reported that arsenic interfered with transcription factors involved in insulin-related gene expression: upstream factor 1 in pancreatic β-cells and peroxisome proliferative-activated receptor γ in preadipocytes. Other *in vitro* studies assessed the effect of arsenic on glucose uptake, typically using very high concentrations of arsenite or arsenate. These studies provide limited insight on potential mechanisms. We identified 10 *in vivo* studies in animals. These studies showed inconsistent effects of arsenic on glucose metabolism. Finally, we identified 19 epidemiologic studies (6 in high-arsenic areas in Taiwan and Bangladesh, 9 in occupational populations, and 4 in other populations). In studies from Taiwan and Bangladesh, the pooled relative risk estimate for diabetes comparing extreme arsenic exposure categories was 2.52 (95% confidence interval, 1.69–3.75), although methodologic problems limit the interpretation of the association. The evidence from occupational studies and from general populations other than Taiwan or Bangladesh was inconsistent. In summary, the current available evidence is inadequate to establish a causal role of arsenic in diabetes. Because arsenic exposure is widespread and diabetes prevalence is reaching epidemic proportions, experimental studies using arsenic concentrations relevant to human exposure and prospective epidemiologic studies measuring arsenic biomarkers and appropriately assessing diabetes should be a research priority.

Type 2 diabetes mellitus is a metabolic disorder characterized by hyperglycemia, insulin resistance in peripheral tissues, and altered insulin secretory capacity of pancreatic β-cells. Type 2 diabetes accounts for 90–95% of all cases of diabetes and is a major public health problem worldwide (Wild et al. 2004). Established risks factors of type 2 diabetes include older age, obesity, physical inactivity, family history, and genetic polymorphisms. In addition, environmental toxicants, including arsenic, have been suggested to play an etiologic role in diabetes development (Longnecker and Daniels 2001).

Arsenic is a recognized toxicant and carcinogen. Nonoccupational exposure occurs mainly through drinking water, affecting millions of people worldwide. Exposure to levels of arsenic in drinking water well above 100 ppb has been associated with an increased risk of type 2 diabetes in the high-arsenic areas of Taiwan and Bangladesh (Lai et al. 1994; Rahman et al. 1998). The biological mechanisms for an association between chronic arsenic exposure and increased diabetes risk are not known [National Research Council (NRC) 1999, 2001; Tseng 2004].

Previous reviews of the role of arsenic in diabetes have questioned the quality of the evidence but were supportive of the possibility of an association [NRC 1999, 2001; Ng 2001; Tseng 2004; Tseng et al. 2000, 2002; World Health Organization (WHO) 2001]. These reviews, however, did not use systematic review criteria and may be subject to biased selection of the evidence. Our objective was to perform a systematic review of the experimental and epidemiologic evidence on arsenic and type 2 diabetes. We examined experimental studies (*in vitro* or *in vivo*) to synthesize available information on plausible mechanisms for the effect of arsenic on glucose metabolism, as well as epidemiologic studies to synthesize the association of arsenic exposure with diabetes risk in humans.

## Materials and Methods

### 

#### Search strategy and study selection.

We searched the Medline database (http://www.ncbi.nlm.nih.gov/entrez/query.fcgi?db=PubMed) and the TOXNET database [consisting of TOXLINE, GENETOX, and DART/ETIC (Developmental and Reproductive Toxicology/Environmental Teratogen Information Center); http://toxnet.nlm.nih.gov/] from 1966 through July 2005 using free text and the medical subject headings (MeSH) arsenic, arsenite, arsenate, arsenicals, diabetes, glucose, glycosylated hemoglobin, insulin, and mortality. In addition, we manually reviewed the reference lists from relevant original research and review articles.

For experimental studies, we identified *in vitro* or *in vivo* studies of the administration of arsenic or arsenic compounds, including inorganic arsenite (trivalent arsenic), inorganic arsenate (pentavalent arsenic), and others, and outcomes related to diabetes status or glucose and insulin metabolism. For epidemiologic studies, we identified studies assessing arsenic exposure through measures of environmental samples, biomarkers, or indirect measures (e.g., job titles reflecting occupational exposure or living in areas with known exposure via drinking water) and diabetes status or markers of glucose metabolism.

**Figure 1:**
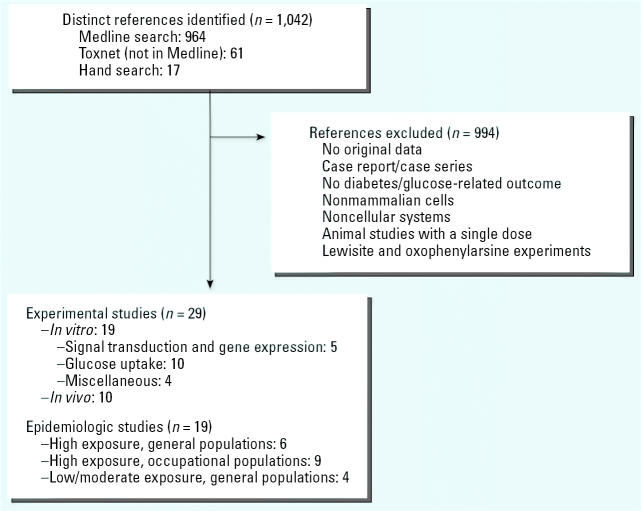
Flow diagram of the experimental and epidemiologic study selection process.

The exclusion criteria for experimental and epidemiologic studies were *a*) no original research (reviews, editorials, nonresearch letters); *b*) studies performed only on people with diabetes, including case reports; *c*) lack of outcomes related to diabetes or glucose metabolism; *d*) no data on arsenic exposure; *e*) experiments in nonmammalian cells, or noncellular experiments; *f*) animal studies administering a single dose of arsenic; and *g*) culture cell experiments using lewisite or oxophenylarsine. Figure 1 summarizes the study selection process.

Two investigators (A.N.-A., R.A.S.) independently abstracted the articles that met the selection criteria. Discrepancies were resolved by consensus. We converted all arsenic concentrations to parts per million (ppm) or parts per billion (ppb), including concentrations from *in vitro* studies, which were usually reported in molar units of arsenic (1 μM of arsenic = 74.9 μg/L = 74.9 ppb).

#### Statistical methods.

Measures of association in epidemiologic studies (odds ratios, prevalence ratios, standardized mortality ratios, relative risks, relative hazards, comparisons of means) and their SE values were abstracted or derived using data reported in the articles (Greenland 1987). Within each study, we used the model adjusted for the most covariates. Adjustment did not substantially modify the conclusions of any individual study. For five studies, we used data available in the original articles to derive relative risk estimates. For one study (Lagerkvist and Zetterlund 1994), because there were no cases among the unexposed, we added 0.5 to each cell to estimate the relative risk and the 95% confidence interval (CI). For Jensen and Hansen (1998), we compared the proportion of subjects with glycosylated hemoglobin above 7% across occupational exposure categories. For Ward and Pim (1984) and Ruiz-Navarro et al. (1998), we used the linear discriminant function method to estimate relative risks from comparisons of means (Greenland 1987). Finally, for Lewis et al. (1999), we estimated the relative risk of diabetes mortality comparing the highest with the lowest category of exposure within the cohort from the published standardized mortality ratios.

We grouped the studies in three categories: studies in general populations exposed to high arsenic levels, corresponding to studies in Taiwan and Bangladesh with average levels in drinking water well above 100 ppb; studies in occupational populations exposed to high arsenic levels most commonly in ambient air; and studies in general populations exposed to low or moderate levels of arsenic in drinking water (< 100 ppb), food, or ambient air. Because of substantial heterogeneity and methodologic limitations, we present a qualitative systematic review, and we used only meta-analysis techniques for studies from Taiwan and Bangladesh. For descriptive purposes, we report the range and the unweighted medians of the relative risk of diabetes comparing extreme categories of arsenic exposure in each study.

## Results

### *In Vitro* Experimental Studies

Nineteen *in vitro* studies published between 1965 and 2004 met our inclusion criteria (Figure 1, Table 1). None of the experimental studies were conducted in human cell lines. Five experiments investigated the effect of arsenic on insulin signal transduction and gene expression. Three studies were performed in transfected mouse pancreatic β-cells, where exposure to high arsenite concentrations was similar to high glucose in stimulating insulin upstream factor 1 (IUF-1) (Macfarlane et al. 1997) and in stimulating the translocation of IUF-1 from the cytoplasm to the nucleus (Elrick and Docherty 2001; Macfarlane et al. 1999). IUF-1, also called homeodomain transcription factor PDX1, is a transcription factor that binds to the human insulin gene promoter and increases insulin messenger RNA levels in response to glucose. The effect of high glucose or arsenite was prevented by SB 203580, a specific inhibitor of stress-activated protein kinase-2 (SAPK2)/p38, whereas the effect of high glucose but not of arsenite was prevented by substances that specifically inactivate phosphatidylinositol-3 kinase (wortmannin and LY294002). Two other studies (Salazard et al. 2004; Wauson et al. 2002) investigated the role of arsenite in adipocyte differentiation and peroxisome proliferative-activated receptor γ (PPARγ) expression. PPARγ is a transcription factor that regulates key gene expression for insulin sensitivity. These two experiments used different concentrations and lengths of exposure and produced opposite results. In the study by Salazard et al. (2004), the incubation of 3T3-F442A preadipocytes with 1.7 and 3 ppb (0.25 and 0.5 μM) arsenite for 3 days induced the expression of PPARγ and CCAAT/enhancer binding protein. In study by Wauson et al. (2002), the incubation of C3H 101T1/2 cells with 450 ppb (6 μM) arsenite for 2 months prevented adipocyte differentiation through the inhibition of the PPARγ. Arsenite also inhibited the differentiating effect induced by pioglitazone, a PPARγ agonist used to reduce insulin resistance.

**Figure 2:**
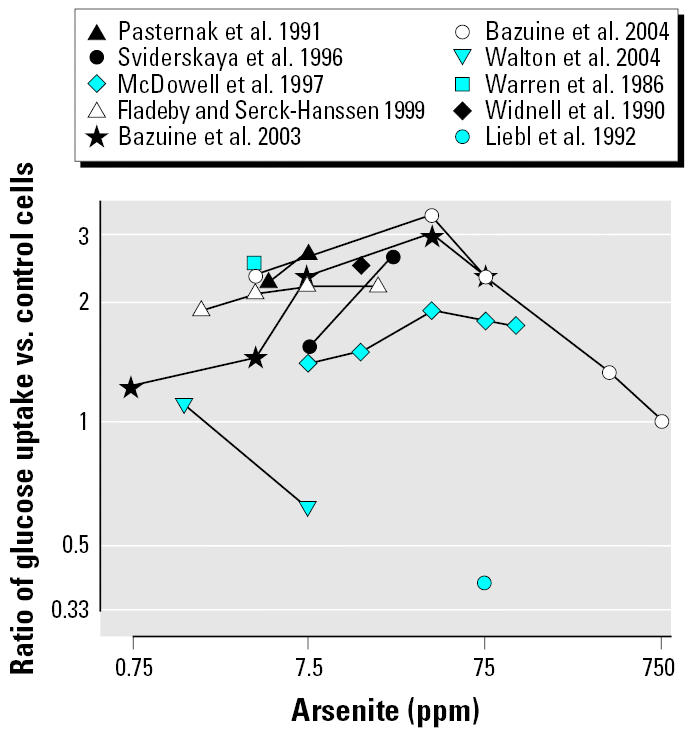
Ratio of basal glucose uptake in peripheral cell lines comparing arsenite versus control. Lines represent the dose response for each independent study. Single points represent the effect for studies using a single dose (1 ppm = 13.35 μM; 0.75 ppm = 10 μM).

The rest of the *in vitro* studies assessed the effect of arsenic on glucose uptake, typically using very high concentrations of arsenite as general inducers of cellular stress. Ten studies measured basal glucose uptake (in the absence of insulin) in cell lines exposed to arsenite or other compounds (Table 1, Figure 2). Four of the studies also exposed the cells simultaneously to insulin and arsenite (Table 2). Compared with insulin alone, simultaneous exposure to insulin and arsenite decreased glucose uptake in insulin-sensitive cells (Bazuine et al. 2003; Walton et al. 2004). One of the studies (Walton et al. 2004) measured basal and insulin-stimulated glucose uptake in cells exposed to arsenate and to methylated arsenic compounds. Methylarsine oxide (MAs^III^O) inhibited insulin-stimulated glucose uptake at the concentration of 75 ppb after 4- or 24-hr exposure (Walton et al. 2004). For arsenite, because the concentrations used in glucose uptake studies were extremely high, their relevance to diabetes development in humans is questionable.

Overall, *in vitro* studies provided limited insight into potential mechanisms that may explain an etiologic role of arsenic on diabetes.

### *In Vivo* Experimental Studies

Ten experimental studies in mice, rats, goats, and guinea pigs published between 1979 and 2004 met our inclusion criteria (Figure 1, Table 3). Arsenite was evaluated in 6 studies (Biswas et al. 2000; Cobo and Castineira 1997; Ghafghazi et al. 1980; Pal and Chatterjee 2004a, 2004b, 2005), and arsenate in 2 studies (Aguilar et al. 1997; Hughes and Thompson 1996). Other compounds were methanearsonate (Judd 1979) and monomethylarsenic (Arnold et al. 2003). Six studies administered arsenic in water or food for lengths of time ranging from 4 weeks to 2 years, and 5 studies involved intraperitoneal exposure to arsenic for 5–30 days. The doses of arsenic were high or very high in most studies, with a lowest dose of 5.55 ppm arsenite (Pal and Chatterjee 2004a) and 0.025 ppm arsenate (Hughes and Thompson 1996).

Although all studies measured glucose levels in blood, plasma, or serum, only one study provided information on potential mechanisms (Cobo and Castineira 1997). In this study, the oral administration of arsenite did not affect insulin levels *in vivo*. However, a glucose stimulus applied *ex vivo* produced greater insulin release from the isolated pancreas cells of rats treated with arsenite *in vivo* compared with the insulin release from isolated pancreas cells of control rats.

### Epidemiologic Studies

#### Study characteristics.

Nineteen epidemiologic studies met our inclusion criteria (Figure 1, Table 4). Three studies were published between 1980 and 1984 (Enterline and Marsh 1982; Mabuchi et al. 1980; Ward and Pim 1984), and the other 15 were reported between 1994 and 2004. Only 2 studies used a prospective cohort design (Lewis et al. 1999; Wang et al. 2003). The rest used cross-sectional, case–control, or retrospective cohort designs. Two studies used the WHO diabetes definition based on oral glucose tolerance tests and/or self-reported medication to define diabetes, whereas the other studies used death certificates, medical or insurance records, urine tests for glucosuria, self-reported diabetes symptoms such as polyuria confirmed by two positive urine tests and a positive oral glucose tolerance test, glycosylated hemoglobin, or self-reported diagnosis. Two studies did not specify the diagnostic criteria. The number of diabetes cases ranged from 2 (Mabuchi et al. 1980) to 27,543 (Wang et al. 2003), but most studies had fewer than 100 cases. Studies in general populations included adult men and women, whereas occupational studies included mostly men.

There were substantial differences in arsenic exposure ascertainment. Most studies in general populations assessed exposure indirectly, using measurements of total arsenic levels in community drinking water sources. Two studies from Taiwan (Lai et al. 1994; Tseng et al. 2000), one from Bangladesh (Rahman et al. 1999), and one from the United States (Lewis et al. 1999) estimated a cumulative arsenic exposure index (ppm-year) by multiplying the number of years that individuals lived in a specific village/area by the average arsenic level in drinking water in that village/area (usually, in each area, several measurements were performed once in time). Other studies in Taiwan and Bangladesh assigned exposure on the basis of residence in an area determined to be endemic for arseniasis (Rahman et al. 1998; Tsai et al. 1999; Wang et al. 2003). None of the studies from Taiwan or Bangladesh obtained individual measures of arsenic exposure either from household tap water measures or more directly by using biomarkers of exposure. None of these studies assessed potential sources of exposure other than drinking water. In occupational studies, exposure was based on job title or on estimated arsenic levels in air for different job categories as assessed by a safety engineer (Rahman and Axelson 1995). One study in an occupationally exposed area assessed arsenic exposure based on years of residence within 4 km of a copper smelter during childhood (Tollestrup et al. 2003). Some occupational studies (Enterline and Marsh 1982; Jensen and Hansen 1998; Lagerkvist and Zetterlund 1994; Lubin et al. 2000) also measured arsenic in urine or air to confirm exposure, but this information was not linked to diabetes in the analyses. Only two studies used biomarkers of exposure: Ward and Pim (1984) measured total arsenic in plasma, and Ruiz-Navarro et al. (1998) measured total arsenic in urine, without speciation of inorganic and methylated compounds.

#### Quality assessment.

In the epidemiologic studies we abstracted information to evaluate study quality, adapting the criteria proposed for observational studies by Longnecker et al. (1988). As shown in Table 5, most studies failed to fulfill important quality criteria such as individual measures of arsenic exposure using biomarkers, standard criteria to diagnose diabetes, or information on established risk factors for diabetes.

#### Relative risk estimates.

The relative risk estimates comparing the highest with the lowest arsenic exposure categories are shown in Table 4. Studies in Taiwan and Bangladesh consistently identified an increased risk of diabetes with increased arsenic exposure, with relative risks ranging from 1.46 to 10.1 (median, 2.40) and with a pooled relative risk estimate using and inverse variance weighted random-effects model of 2.52 (95% CI, 1.69–3.75; *p* heterogeneity < 0.001). Occupational studies were small and showed no consistent pattern, with relative risks ranging from 0.34 to 9.61 (median, 1.40). We identified only 4 studies in general populations from countries with low or moderate arsenic exposure. These studies were small and did not show an increased risk of diabetes with increasing arsenic levels (relative risks ranged from 0.65 to 1.09; median, 0.95).

**Figure 3:**
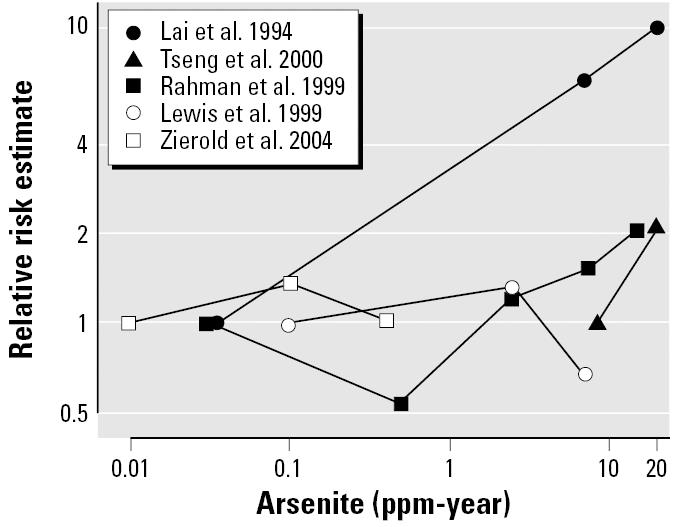
Risk of diabetes by cumulative arsenic exposure in drinking water in epidemiologic studies. Black lines represent the dose response for studies in Taiwan and Bangladesh compared with the baseline category of exposure. Gray lines represent the dose response in studies in the United States. Cumulative exposure: ∑ arsenic levels in drinking water*_i_* × time of exposure***i*** (*i* indicates specific village). For example, a cumulative exposure of 1 ppm-year is reached after 10 years of residence in a village with an arsenic concentration in drinking water of 0.1 ppm. In the study by Zierold et al. (2004), we assumed 20 years of exposure for all study subjects.

Five studies provided information on the dose response in diabetes risk by cumulative arsenic exposure in drinking water (Figure 3). Diabetes risk tended to increase with increasing cumulative exposure in studies from Taiwan (Lai et al. 1994; Tseng et al. 2000) and Bangladesh (Rahman et al. 1999). No trend was observed in the U.S. studies (Lewis et al. 1999; Zierold et al. 2004).

## Discussion

### 

#### Summary of findings.

The evidence on the association of arsenic exposure with diabetes risk summarized in this systematic review is inconclusive. Evidence from *in vitro* studies suggests that arsenic interferes with signal transduction and transcription factors that are related to insulin pathways such as IUF-1 in pancreatic cells or PPARγ in preadipocytes. *In vitro* glucose uptake experiments and *in vivo* studies did not provide evidence on potential mechanisms that may explain a diabetogenic effect of arsenic. In general, experimental studies were limited by the use of arsenic concentrations that were much higher than those relevant to human exposure. For example, the current U.S. Environmental Protection Agency recommended standard for arsenic in drinking water is 10 ppb. The lowest concentration of arsenite used in studies of cultured cells investigating glucose uptake was 750 ppb (Bazuine et al. 2003), and the lowest concentration of arsenite in animal studies was 5,550 ppb (Pal and Chatterjee 2004a, 2004b).

In epidemiologic studies, the association between arsenic exposure and diabetes across different populations and different sources of exposure was inconsistent. In populations exposed to high arsenic levels via drinking water in Taiwan and Bangladesh, diabetes risk was consistently increased. In occupational settings, diabetes mortality was increased in some studies and decreased in others. Finally, no association with diabetes was observed in four studies of general populations outside of Taiwan or Bangladesh. Overall, the quality of the epidemiologic evidence was limited by methodologic problems, particularly in assessing arsenic exposure and diabetes outcomes.

#### Mechanisms for arsenic-related diabetes.

Acute arsenite toxicity, including its effects on glucose metabolism, is generally attributed to its reactivity toward thiol (SH) groups (Aposhian 1989; NRC 1999). During acute poisoning, arsenite inhibits pyruvate and α-ketoglutarate dehydrogenases (Aposhian 1989), essential enzymes for gluconeogenesis and glucolysis. The interference of arsenic with pyruvic acid metabolism was described by Krebs in the early 1930s (Krebs 1933). Arsenate, on the other hand, can replace phosphate in energy transfer pathways of phosphorylation and also uncouples oxidative phosphorylation (Kennedy and Lehninger 1949). However, these toxic effects of acute arsenic exposure are unlikely to occur as a result of chronic exposure to environmentally relevant doses (Tseng 2004).

The influence of arsenic on the expression of gene transcription factors may be related to diabetes risk. However, the effects of arsenite on IUF-1 and PPARγ were contradictory in terms of diabetes development. The differential effects may reflect a complex dose–response pattern for arsenic or differences in length of exposure to arsenic across studies. Further studies with wide ranges and durations of arsenic exposure are needed to investigate the effect of arsenic on these and other insulin-related events at the cellular and molecular levels. For instance, interference with the glucocorticoid receptor is another potential mechanism for arsenic-related diabetes that deserves further investigation. Arsenic shows a complex dose–response effect on glucocorticoid receptor mediated transcription (Bodwell et al. 2004), with a stimulatory effect at very low concentrations (6–120 ppb) and an inhibitory effect at doses greater than 120 ppb. The glucocorticoid receptor is a member of the steroid receptor superfamily that among other metabolic processes regulates gluconeogenesis. Reduction of glucocorticoid receptor expression in hepatic and adipose tissue has been shown to improve hyperglycemia in diabetic rodents (Watts et al. 2005).

Experimental studies on glucose uptake showed that arsenite increases uptake independently of the earlier steps of the insulin transduction pathway, although when co-administered with insulin, arsenite inhibited insulin-stimulated glucose uptake in 3T3-L1 adipocytes. The purpose of most of these studies was to investigate the role of stress in glucose uptake, which is unrelated to the possibility that arsenic could affect diabetes risk. Under these designs, cultured cells were exposed to high arsenic levels for a few hours, whereas humans are chronically exposed to lower concentrations. Only one study investigated methylated arsenical compounds and their interference in insulin signaling in adipocytes (Walton et al. 2004). For these reasons, the relevance of *in vitro* glucose uptake findings to diabetes etiology is uncertain.

Arsenic could influence diabetes development by other mechanisms, including oxidative stress, inflammation, or apoptosis, nonspecific mechanisms that have been implicated in the pathogenesis of type 2 diabetes. Arsenic exposure can enhance the production of reactive oxygen species (Barchowsky et al. 1999; Chen et al. 1998; Tseng 2004; Wang et al. 1996), interfere with the activity of key antioxidant enzymes such as glutathione reductase, glutathione *S*-transferase, glutathione peroxidase, and glucose 6-phosphate dehydrogenase (Maiti and Chatterjee 2000; Santra et al. 2000), and induce lipid peroxidation (Santra et al. 2000). In individuals from Taiwan, increasing blood levels of arsenic correlated with increasing levels of reactive oxygen species and with decreasing levels of antioxidant capacity in plasma (Wu et al. 2001). Arsenic may also up-regulate interleukin-6 and other inflammatory cytokines (Wu et al. 2003), and it may induce the release of tumor necrosis factor-α from mononuclear cells (Yu et al. 2002). Finally, arsenic is well known for inducing apoptosis in multiple cell lines (Waalkes et al. 2000). Future research should evaluate whether these mechanisms mediate the role of arsenic in diabetes development.

The *in vivo* experimental studies were mostly uninformative. The diversity of species studied probably reflects that there are no good animal models to study the effects of arsenic on diabetes development. Indeed, the classification of arsenic as a human carcinogen, although recently supported by animal models (Waalkes et al. 2004), was for a long time based on human data. Progress in the study of the role of arsenic in diabetes requires the identification of appropriate animal models.

#### Arsenic and diabetes in human studies.

Suggestive evidence links chronic exposure to high arsenic levels in drinking water with increased diabetes risk in Taiwan and Bangladesh. Methodologic problems, however, limit the causal interpretation of this association. The use of average drinking water and the lack of individual measures of arsenic make it possible to underestimate exposure due to between-subject variability in water consumption and to other sources of arsenic exposure in these areas, such as contaminated food and cooking water. On the other hand, because arsenic exposure was assessed at the village level and diabetes diagnosis was often not performed according to standard procedures, this ecologic association could reflect the uncertain comparability of exposure groups in terms of socioeconomic development, access to care, study selection factors and other diabetes risk factors. The use of urine tests and of administrative data to identify diabetes makes it likely that only severe or symptomatic cases were identified, and it is uncertain whether the procedures and frequency for diabetes testing were similar across areas with different arsenic exposure. In addition, the use of administrative data can be affected by surveillance and diagnostic biases. For example in Taiwan, arsenic-related health problems in the endemic areas are well known, hence, subjects in these areas may have received different medical care, including different diagnostic services, compared with subjects in areas with lower arsenic levels.

It is also possible that the findings from Taiwan and Bangladesh may not be generalizable to other populations. Some reasons for this include variations in the distribution of polymorphisms in genes involved in arsenic metabolism or response (Loffredo et al. 2003), differences in arsenic species to which populations were exposed (Chen et al. 1995), other co-exposures (Chen et al. 1995), and dietary deficiencies that may interact with arsenic. For example, selenium and zinc levels in Taiwan and Bangladesh are among the lowest worldwide (Lin and Yang 1988), and poor dietary selenium has been suggested as an underlying factor for arsenic and cancer in Bangladesh and West Bengal in India (Spallholz et al. 2004). In guinea pigs, selenium and arsenic counteract each other in glucose metabolism (Das et al. 1989), and the joint effect of high arsenic and low selenium could play a role in diabetes development. Exposure to arsenic, selenium, other nutrients, and other diabetes risk factors were not measured in epidemiologic studies.

We found no reports of diabetes in populations known to be exposed to high levels of arsenic in drinking water in Chile and Argentina. This lack of information on diabetes could reflect a lack of research, but it has also been suggested to be related to publication bias (Longnecker and Daniels 2001).

The evidence from general populations outside of Taiwan or Bangladesh was inconclusive because of the small number of cases, limitations in study design, and misclassification of diabetes status. Occupational studies, on the other hand, could not be interpreted in favor or against an association because of uncertain comparability of study participants with the general population used as reference, limitations in exposure assessment, lack of information on concomitant exposures, lack of information on major diabetes risk factors, and the possibility of a healthy worker survivor effect.

An important conclusion we derived from the epidemiologic review is the limited quality of the evidence base. This finding is consistent with previous reviews, including those by U.S. and international panels (NRC 1999, 2001; Ng 2001; WHO 2001). These panels determined that the available evidence on arsenic and diabetes suffered from uncertainties in study design and exposure assessment. Our review further refines these reports and identifies the lack of biomarker data and the lack of standard criteria for diabetes assessment as major limitations of the evidence base. Current uncertainties in the role of arsenic in diabetes development could be reduced by conducting carefully planned epidemiologic studies in populations exposed to a wide range of arsenic levels. Future studies should *a*) measure appropriate arsenic biomarkers that integrate all sources of exposure (e.g., urine or toenails); *b*) carefully collect information on current and past sources of arsenic exposure and on potential confounders and modifiers, including known determinants of diabetes development; *c*) and prospectively ascertain diabetes using standard definitions.

## Conclusion

The possibility of an association between chronic arsenic exposure and diabetes has implications for research and public health. Millions of people are exposed worldwide to moderate or high levels of arsenic in drinking water. Because diabetes is also a major public health problem, the public health consequences of a causal association could be serious. Methodologic problems limit the causal interpretation of the moderately strong association between high arsenic exposure and diabetes in Taiwan and Bangladesh. Overall, the experimental and epidemiologic evidence is at present insufficient and inadequate to establish causality. Experimental studies that use arsenic concentrations relevant to human exposures, and high-quality prospective epidemiologic studies that use appropriate methods for exposure assessment as well as rigorous criteria for outcome definitions should be research priorities.

## Correction

Table 1 has been modified from the original manuscript published online. The table now includes information on the 24-hr study by Walton et al. (2004).

## Figures and Tables

**Table 1:** *In vitro* studies of arsenic exposure and glucose metabolism outcomes.

Source	Type of cell/tissue	Compound	Dose (ppm)	Incubation	Outcomes and results (compared with controls)
Signal transduction and gene expression					
Macfarlane et al. 1997	Pancreatic β-cells	Arsenite	37.5	0.33 hr	↑ IUF-1 dependent gene expression PI-3 kinase independent; SAPK2/p38 involved
Macfarlane et al. 1999	Pancreatic β -cells	Arsenite	75	0.5 hr	↑ IUF-1 translocation from cytoplasm to nucleus PI-3 kinase independent; SAPK2/p38 involved
Elrick and Docherty 2001	Pancreatic β-cells	Arsenite	75	0.5 hr	↑ IUF-1 translocation from cytoplasm to nucleus PI-3 kinase independent; SAPK2/p38 involved
Wauson et al. 2002	C3H 10T1/2 preadipocytes	Arsenite	0.45	2 months	↓ PPARγ mRNA ↓ Pioglitazone-stimulated adipocyte differentiation
Salazard et al. 2004	3T3-F442A preadipocytes	Arsenite	0.0017, 0.003	3 days	↑ Expression of PPARγ and C/EBPα(genes with important roles in adipose determination)
Glucose uptake in cultured cells					
Warren et al. 1986	BHK-21 cells	Arsenite	3.75	2 hr	↑ Basal glucose uptake; = insulin-stimulated glucose uptake = amino acid uptake
Widnell et al. 1990	BHK-21 cells	Arsenite	15	2 hr	↑ Basal glucose uptake; ↑ glucose transporter translocation (reversible)
Pasternak et al. 1991	BHK-21 cells	Arsenite	4.5–7.5	2 hr	↑ Basal glucose uptake (reversible when arsenite removed) Fast and reversible translocation of glucose receptor
Liebl et al. 1992	MDCK dog cells	Arsenite	37.5–75	1 hr	↓ Basal glucose uptake, dose dependent
Sviderskaya et al. 1996	BHK cells 3T3-L1 adipocytes	Arsenite	7.5–22.5	2 hr	↑ Basal glucose uptake, dose dependent ↑ Glucose transporter translocation in both types of cells
McDowell et al. 1997	L6 rat muscle cells	Arsenite	7.5–112.5	0.5 hr	↑ Basal glucose uptake, dose dependent but maximal with 37.5 ppm ↑ GLUT1 and GLUT4 in cell membrane, PI-3 kinase independent ↑ Insulin-stimulated glucose uptake
Fladeby and Serck-Hanssen 1999	Bovine adrenal cells	Arsenite	1.88–18.8	1 hr	↑ Basal glucose uptake up to 7.5 ppm, then plateau PI-3 kinase independent, SAPK2/p38 partly involved
Bazuine et al. 2003	3T3-L1 adipocytes	Arsenite	0.75–75	0.5 hr	↑ Basal glucose uptake up to 37.5 ppm, then ↓ ↓ Insulin-stimulated glucose uptake ↑ GLUT4 and GLUT1 translocation (but less than insulin) PI-3 kinase independent; no changes in IRβ, IRS-1, IRS-2 No phosphorylation of PKB; PKC-λ/ζ and SAPK2/p38 involved
Bazuine et al. 2004	3T3-L1 adipocytes	Arsenite	3.75–750	0.5 hr	↑ Basal glucose uptake up to 37.5 ppm Dexamethasone ↓arsenite glucose uptake SAPK2/p38 involved
Walton et al. 2004	3T3-L1 adipocytes	Arsenite	1.5, 7.5	4 hr	= basal glucose uptake at 1.50 ppm, ↓at 7.5 ppm, ↓insulin-stimulated
		MAs^III^O	0.08, 0.4		= basal glucose uptake at 0.08 ppm, ↓at 0.4 ppm, ↓insulin-stimulated
		DMAs^III^I	0.15, 0.75		= basal glucose uptake all doses, ↓insulin-stimulated
		Arsenate	7.5, 75		↑ basal glucose uptake at 7.5 ppm, ↓at 75 ppm, = insulin-stimulated
		MAs^V^	7.5, 75		= basal glucose uptake all doses, ↓insulin-stimulated
		DMAs^V^	7.5, 75		= basal and insulin-stimulated glucose uptake all doses, PI-3 kinase independent. No changes in IRβand IRS-2 MAs^III^O and DAs^III^I, but not arsenite IRS-1, ↑phosphorylation of IRS-1 Arsenite, MAs^III^O and DAs^III^I ↓phosphorylation of PKB/Akt Arsenite, MAs^III^O and DAs^III^I ↓GLUT4 translocation in insulin-treated cells
		Arsenite	0.4, 0.8, 1.5	24 hr	Dose-dependent ↓insulin-stimulated glucose uptake
		MAs^III^O	0.02, 0.04, 0.08		= insulin-stimulated glucose uptake at 0.02 ppm, ↓at 0.04 and 0.08
		DMAs^III^I	0.04, 0.08, 0.15		= insulin-stimulated glucose uptake all doses
Miscellaneous experiments					
Short et al. 1965	Rat hemidiaphragms	Arsenite	75	1–3 hr	↑ Basal glucose uptake in hemidiaphragms; ↑uptake with arsenate in fat pads = insulin stimulated glucose uptake in hemidiaphragm; ↓ uptake with arsenite in fat pad
	Epidydimal fat pads	Arsenate	75		
Dixit and Lazarow 1967	Epidydimal fat pads	Arsenite	0.75–7,500	3 hr	↑ Basal glucose oxidation up to 7.5 ppm
Brazy et al. 1980	Rabbit kidney tubules	Arsenate	0.75–375	0.5 hr	↓ Fluid, phosphate, and glucose absorption (lumen to bath)
Hunder et al. 1993	Rat jejunal segments	Arsenite	0.19–18.9	2 hr	↓ Intestinal glucose transfer dose dependent (= arsenate < 7.5 ppm)
		Arsenate	0.19–187.5		

1 ppm = 13.35 μM. Basal glucose uptake, glucose uptake in the absence of insulin. ↑, increase; ↓, decrease; = similar levels; BHK-21 cells, baby hamster kidney cells (contain predominantly GLUT1); C/EBPα, CCAAT/enhancer binding protein; DAs^III^I, iododimethylarsine; DMAs^V^, dimethylarsinic acid; GLUT, glucose transporter; IRβ: insulin receptor β; IRS, insulin receptor substrate; IUF-1, insulin upstream factor-1 (also called homeodomain transcription factor PDX1); MAs^III^O, methylarsine oxide; MAs^V^, monosodium methyl arsenate; MDCK dog cells, Madin-Darby canine kidney cells; ppm, part per million; PI-3 kinase, phosphatydilinositol-3 kinase; PKB, protein kinase B; PKC, protein kinase C; PPARγ, peroxisome proliferative-activated receptor γ; SAPK2, stress activator protein kinase 2 (also called p38 mitogen-activated protein kinase).

**Table 2:** Experimental characteristics and ratio of glucose uptake in peripheral cell lines exposed to arsenite and insulin compared with insulin and arsenite alone.

	Experiment characteristics				Ratio of glucose uptake vs.	
Source	Type of cell	Incubation (hr)	Arsenite (ppm)	Insulin (nM)	Insulin	Arsenite
Warren et al. 1986	BHK-21 cells	2	3.75	100	0.94	0.91
McDowell et al. 1997	L6 rat muscle cells	0.5	37.5	100	1.42	1.21
Bazuine et al. 2003	3T3-L1 adipocytes	0.5	37.5	100	0.57	1.33
Walton et al. 2004	3T3-L1 adipocytes	4	1.50	1,000	0.60	0.55
Walton et al. 2004	3T3-L1 adipocytes	4	7.49	1,000	0.20	0.33

BHK-21 cells, baby hamster kidney cells. For arsenite, 1 ppm = 13.35 μM.

**Table 3:** *In vivo* studies of arsenic exposure and glucose metabolism.

Source	Experimental Animal	*n*	Compound (route)	Daily dose (ppm)	Duration	Outcomes and results (compared with controls)
Judd 1979	Field mice	19	Methanearsonate (po in water)	1,000	30 days	↓ Blood glucose, = fluid and food consumption
Ghafghazi et al. 1980	Rats	12	Arsenite (ip)	5–10	7 days	↑ Glucose levels after glucose tolerance test, dose dependent
Hughes and Thompson 1996	B6C3F_1_ mice	72	Arsenate (po in water)	0.025–2.5	28 days	↓ Plasma glucose, = fluid and food consumption
Aguilar et al. 1997	Wistar rats	20	Arsenate (po in food)	5	10 weeks	= Plasma glucose levels
Cobo and Castineira 1997	Wistar rats	21	Arsenite (po in water)	17.75	1st week	Delayed glucose clearance after glucose tolerance test
				up to 100	8th week	= Basal insulin levels *in vivo*
Biswas et al. 2000	Bengal goats	12	Arsenite (po in capsule)	25	12 weeks	↑ Blood glucose at week 6 and ↑↑ at week 12
Arnold et al. 2003	Fischer rats	480	Monomethylarsenic (po in food)	50–1,300	2 years	= Blood glucose levels up to 400 ppm, ↓with 1,300 ppm
Pal and Chatterjee 2004a	Wistar rats	18	Arsenite (ip)	5.55	21 days	↓ Blood glucose (reversed with methionine) = Body, liver, kidney weight
Pal and Chatterjee 2004b	Wistar rats	18	Arsenite (ip)	5.55	30 days	↓ Blood glucose (reversed with *N*-acetylcysteine)
Pal and Chatterjee 2005	Wistar rats	18	Arsenite (ip)	5.55	30 days	↓ Blood glucose (reversed with methionine)

Abbreviations: ip, intraperitoneal; po, per oral; ↑, increase; ↓, decrease.

**Table 4:** Epidemiologic studies of arsenic exposure and diabetes.

Source	Design	Country	Population	Diabetes diagnosis	Cases/noncases	Men (%)	Age range (year)	Arsenic assessment	Levels, exposed vs. reference	RR of diabetes (95% CI)	Adjusted for
General populations, high arsenic exposure											
Lai et al. 1994	CS	Taiwan	Survey of participants in high-arsenic area	OGTT or self-reported	86/805	43	30–69	CEI village drinking water	> 15 vs. 0 ppm-year	10.1 (1.30–77.9)	Age, sex, BMI, physical activity
Tsai et al. 1999	RCO	Taiwan	Deaths in 1971–1994	Death certificate	531 deaths	35	All ages	Living in HAA	HAA vs. no HAA	1.46 (1.28–1.67)	Age, sex
Tseng et al. 2000	CO	Taiwan	Survey of participants in high-arsenic area	OGTT	41/405	50	Mean 47	CEI village drinking water	> 17 vs. < 17 ppm-year	2.10 (1.10–4.20)	Age, sex, BMI
Wang et al. 2003	CS	Taiwan	National Health Insurance Database	ICD-9 250 ICD-9 A181	27,543/678,791	43	25–65+	Living in HAA	HAA vs. no HAA	2.69 (2.65–2.73)	Age, sex
Rahman et al. 1998	CS	Bangladesh	Survey participants in high- and low-arsenic areas	Self-reported symptoms + glucosuria + OGTT	46/971	59	30–60+	Living in HAA and keratosis	Keratosis vs. no keratosis	5.90 (2.90–11.6)	Age, sex, BMI
Rahman et al. 1999	CS	Bangladesh	Survey participants in high-arsenic area	Glucosuria	263/1,332	61	30–60+	CEI village drinking water	> 10 vs. 0 ppm-year	2.10 (1.10–4.20)	Age, sex
Occupational populations, high arsenic exposure											
Mabuchi et al. 1980	RCO	U.S.	Pesticide workers, Baltimore, MD	Death certificate	2 deaths	75	< 20–40+ at hire	Job title	Workers vs. general population	0.47 (0.12–1.88)	Age, sex, period
Enterline and Marsh 1982	RCO	U.S.	Copper smelter workers, Washington State	Death certificate	12 deaths	100	< 20–69 at hire	Job title	Workers vs. general population	0.85 (0.48–1.49)	Age
Lagerkvist and Zetterlund 1994	CS	Sweden	Copper smelter workers, other jobs	Self-reported type 2 diabetes	4/85	100	Mean 57	Job title	Workers vs. other workers	9.61 (0.53–173)	Crude
Rahman and Axelson 1995	CC	Sweden	Copper smelter workers	Death certificate, medical record	12/31	100	30–74 at death	Air levels	~ 5 vs. 0 mg/m^3^	3.30 (0.50–30.0)	Age
Rahman et al. 1996	CC	Sweden	Deaths in glass industry area	Death certificate	240/2,216	100	45–75+	Job title	Workers vs. other workers	1.40 (0.90–2.10)	Age
Jensen and Hansen 1998	CS	Denmark	Taxidermists, wood workers, other jobs	HbA1c	5/59	87	Mean 37	Job title	Workers vs. general population	4.43 (0.47–42.0)	Age
Bartoli et al. 1998	RCO	Italy	Glass industry workers	Death certificate	3 deaths	100	< 40–65+	Job title	Workers vs. general population	0.34 (0.09–0.88)	Age
Lubin et al. 2000	RCO	U.S.	Copper smelter workers, Montana	Death certificate	54 deaths	100	< 20–30+ at hire	Job title	Workers vs. general population	0.83 (0.63–1.08)	Age
Tollestrup et al. 2003	RCO	U.S.	Children < 4 km of Copper smelter	Death certificate	16/3,116	58	< 14	Years of residency	≤10 vs. < 1 year	1.60 (0.36–1.16)	Crude
General populations, low to moderate arsenic exposure											
Ward and Pim1984	CC	UK	Hospital based	NR	87/30	65	18–78	Plasma levels (NAA)	75th vs. 25th percentile	1.09 (0.79–1.49)	Crude
Ruiz-Navarro et al. 1998	CC	Spain	Hospital based	NR	38/49	39	NR	Urinary levels (AAS)	75th vs. 25th percentile	0.87 (0.50–1.53)	Crude
Lewis et al. 1999	CO	U.S.	Mormons	Death certificate	55/4,003	52	< 50–80+	CEI community drinking water	> 4 vs. < 1 ppm-year	0.65 (0.34–1.24)	Age, sex
Zierold et al. 2004	CC	U.S.	Survey participants with private wells	Self-reported	67/1118	NR	Mean 62	Subject drinking water	> 10 vs. < 2 ppb	1.02 (0.49–2.15)	Age, sex, BMI, smoking

Abbreviations: AAS, atomic absorption spectrometry; BMI, body mass index; CC, case–control; CEI, cumulative exposure index: ∑ arsenic levels in drinking water*_i_* *×* time of exposure*_i_* (*i* indicates specific village); CO, cohort; CS, cross-sectional; HAA, high-arsenic area; HbA1c, hemoglobin A1c; ICD-9, *International Classification of Diseases, Ninth revision*; NAA, neutron activation analysis; NR, not reported; OGTT, oral glucose tolerance test, criteria for a positive test based on the WHO criteria; RCO, retrospective cohort; RR, relative risk.

**Table 5:** Criteria for evaluating the design and data analysis of epidemiologic studies on arsenic and diabetes.^a^

	Taiwan and Bangladesh						Occupational populations									Other populations			
	Lai et al. 1994	Tsai et al. 1999	Tseng et al. 2000	Wang et al. 2003	Rahman et al. 1998	Rahman et al. 1999	Mabuchi et al. 1980	Enterline and Marsh 1982	Lagerkvist and Zetterlund 1994	Rahman and Axelson 1995	Rahman et al. 1996	Jensen and Hansen 1998	Bartoli et al. 1998	Lubin et al. 2000	Tollestrup et al. 2003	Ward and Pim 1984	Ruiz-Navarro et al. 1998	Lewis et al. 1999	Zierold et al. 2004
All studies (n = 19)																			
Diabetes diagnosis based on fasting glucose levels or oral glucose tolerance tests	Y	N	Y	N	N	N	N	N	N	N	N	N	N	N	N	N	N	N	N
Exposure assessed at the individual level	N	N	N	N	N	N	N	N	N	Y	N	N	N	N	Y	Y	Y	N	Y
Exposure assessed using a biomarker of exposure	N	N	N	N	N	N	N	N	N	N	N	N	N	N	N	Y	Y	N	N
Control for established diabetes risk factors in addition to age	Y	N	Y	N	N	N	Y	N	N	N	N	N	N	N	N	N	N	N	Y
Case–control and cross-sectional studies (*n* = 11)																			
Response rate among noncases at least 70%^b^	Y	—	—	Y	N	Y	—	—	N	—	—	N	—	—	—	N	N	—	N
Noncases would have been cases if they had developed diabetes	N	—	—	N	N	N	—	—	N	N	N	Y	—	—	—	N	N	—	N
Data collected in a similar manner for all participants	Y	—	—	Y	N	N	—	—	N	Y	Y	Y	—	—	—	N	N	—	Y
Cases interviewed within 6 months of diagnosis	N	—	—	N	N	N	—	—	N	N	N	N	—	—	—	N	N	—	N
Interviewer blinded with respect to the case status of the person interviewed^c^	Y	—	—	—	N	N	—	—	N	N	N	Y	—	—	—	N	N	—	Y
Time period during which all participants were interviewed was the same^c^	Y	—	—	—	N	N	—	—	N	Y	Y	N	—	—	—	N	N	—	Y
Same exclusion criteria applied to all participants	Y	—	—	Y	N	N	—	—	N	Y	N	N	—	—	—	N	N	—	Y
Cohort studies (*n* = 8)																			
Loss to follow-up was independent of exposure	—	N	Y	—	—	—	N	N	—	—	—	—	N	N	N	—	—	Y	—
Intensity of search of disease independent of exposure status	—	N	Y	—	—	—	N	N	—	—	—	—	N	N	N	—	—	Y	—

Abbreviations: —, not applicable; N, no; Y, yes.

a Criteria modified from Longnecker et al. (1988).

b Not applicable to two case–control studies based only on deaths (Rahman and Axelson 1995; Rahman et al. (1996).

c Not applicable to the study using the National Health Insurance Database from Taiwan (Wang et al. 2003).
